# Impact of season on birth weight, growth, and average daily gain of conventionally raised Holstein heifers in the Midwestern United States

**DOI:** 10.3168/jdsc.2025-0758

**Published:** 2025-05-09

**Authors:** K.N. Brost, J.K. Drackley

**Affiliations:** Department of Animal Sciences, University of Illinois, Urbana, IL 61801

## Abstract

The objectives of this study were to determine the effects of season on calf birth weight, growth, and ADG of Holstein heifers. This retrospective analysis included data from July 2018 to February 2022 and included 216 calves from the University of Illinois Dairy Research Unit. Calves were housed in individual hutches until weaned at 56 d. After weaning, calves moved to group housing until 175 d of age, at which time they moved to heifer dry lots. Feeding rates remained consistent throughout the year, and weaning took place from d 50 to 56. Body weight data were collected at birth, weaning, and when moved to heifer lots. Calves were assigned a season based on month of birth: winter (December, January, February), spring (March, April, May), summer (June, July, August), and fall (September, October, November). In this analysis, season was the fixed effect, and calf was a random effect and subject of the repeated effect. Data were analyzed using the MIXED and UNIVARIATE procedures in SAS. Winter-born calves had greater birth and weaning BW when compared with summer calves. Birth BW of winter calves tended to be greater and weaning BW were greater when compared with all nonwinter calves. Similarly, preweaning ADG of winter calves was greater than summer calves and all nonwinter calves. On the other hand, postweaning BW and ADG were greater for summer calves in comparison to winter calves. Likewise, summer calves were observed to have greater BW and tended to have greater ADG in contrast to all nonsummer calves. When combining the pre- and postweaning ADG through the first 175 d of life, there were no differences between winter and summer calves, winter and nonwinter calves, or summer and nonsummer calves. These results indicate calf birth BW and growth performance are greater through weaning during colder seasons. Conversely, calves born during winter do not perform as well after weaning once the seasons get warmer. Summer calves were observed to have decreased birth BW and performance through weaning, but as the seasons became cooler, they excelled postweaning to 175 d of age.

## Highlights

• Winter calves had greater birth and weaning weights in comparison to summer calves.
• Postweaning weights and ADG were greater for summer compared with winter calves.
• Despite birth season and age, all calves performed better during cooler seasons.

## Graphical Abstract

**Figure:**
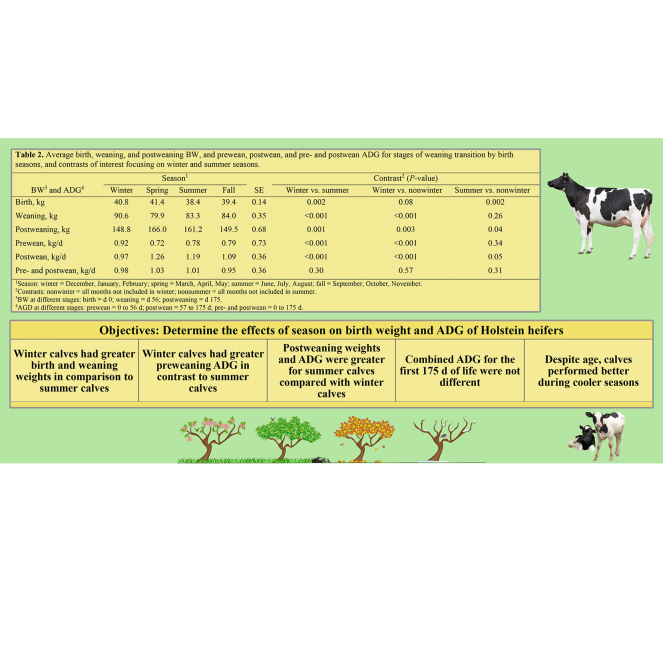
**Summary:** The objectives of this study were to determine the effects of season on calf birth weight, growth, and average daily gain (ADG) of Holstein heifers. This analysis included data from July 2018 to February 2022 and included 216 female calves from the University of Illinois Dairy Research Unit. The results indicate that calf birth weight and growth performance are greater through weaning during the colder winter season. Conversely, calves born during winter do not perform as well after weaning once the seasons get warmer. Summer calves were observed to have decreased birth weight and performance through weaning, but as the seasons became cooler, they excelled postweaning to 175 days of age. These data suggest a need for further research on the impacts of season on calf growth and efficiency, as well as possible lasting effects on future herd performance.

Dairy calves maintain body temperature without using extra energy while in the thermal neutral zone (**TNZ**). The TNZ is 15°C to 25°C for neonates, but changes over the first 21 to 30 d of life to between 0°C to 5°C and 23°C (NRC, 2001; Kohlman and Van Os, 2020). Upper critical temperatures for calves range from 25°C to 32°C, while lower critical temperatures range from 0°C to 10°C, both dependent on environment and animal-based factors (Neuwirth et al., 1979; Drackley, 2008). Heat abatement is seldom considered for calves due to a large surface-to-mass ratio and lower metabolic heat production in comparison to mature cows. Once outside the TNZ, calf metabolic rates rise, attempting to maintain optimal body temperature. Previous studies observed the effect of season on birth BW, specifically greater birth BW during winter and lower birth BW during summer (Place et al., 1998; Bakir et al., 2004). Heat stress can affect thermoregulatory behavior such as panting, sweating, and lying time in preweaning calves (Stull and Raynolds, 2008; Dado-Senn et al., 2022). Further studies show conflicting data on first-lactation milk yields of calves born in differing seasons with both summer- and winter-born heifers having lower first-lactation milk yields in different trials (Chester-Jones et al., 2017; Van Eetvelde et al., 2017, 2020; Fodor et al., 2020). Climactic variations were noted among these studies, indicating a need for further research on seasonal effects on dairy calves. The objectives of this analysis were to determine the effects of season on calf birth BW, growth, and ADG of Holstein heifers. Our hypothesis was that lower birth BW and ADG would be observed for calves born during summer compared with winter. Furthermore, reduced ADG will follow calves born during the summer, possibly leading to reduced performance in the herd. Adapting husbandry based on regional climate might improve calf growth and future herd performance.

Data from 216 female Holstein calves born at the University of Illinois Dairy Research Unit were used for this retrospective analysis. Data from PC Dart (Dairy Records Management Systems, Raleigh, NC) were included from July 2018 to February 2022. Calves were housed in outdoor, south-facing, individual hutches with yards (Calf-tel, Hampel Corp., Germantown, WI). The hutches were bedded with straw and spaced about 1.5 m apart. Calves stayed in the individual hutches until weaned at 56 d of age. Feeding rates were consistent throughout the year with calves offered 950 g/d solids (as-fed), reconstituted to 13% solids with tap water beginning on d 1 after 3 colostrum feedings. From d 1 to 30, calves were fed 3 times daily, and then twice daily from d 31 to 56. Weaning took place from d 50 to 56. Water was offered from d 1 and starter was offered by d 3. Following weaning, calves moved to outdoor group housing until approximately 175 d of age when they were moved to heifer lots. The calf groups were housed in a pasture setting with super hutches (Calf-tel, Hampel Corp., Germantown, WI) and access to a small 3-sided freestall barn, both bedded with straw. The group area was fed starter and TMR through headgates and alfalfa hay from a trough. An automatic waterer was always available. Vaccinations, feeding, and medical treatment were carried out by the farm staff, according to their protocols.

Calves were assigned a season based on month of birth: winter (n = 71; December, January, February), spring (n = 35; March, April, May), summer (n = 50; June, July, August), and fall (n = 62; September, October, November). Body weight data were collected at birth, weaning, and postweaning when moved to the heifer lots by farm personnel. Average daily gain was calculated in Excel (Microsoft Corp.) for preweaning (d 0 to 56 of age), postweaning (d 57 to 175), and the combination of pre- and postweaning.

This analysis was a quasi-experimental design. The experimental unit was the calf. Season was defined as the fixed effect, while calf was the repeated subject and random effect. Least squares means are presented with the largest standard error of the mean. Season comparisons were made with 3 contrasts: winter versus summer, summer versus nonsummer, and winter versus nonwinter, where nonsummer and nonwinter calves include all other seasons not stated in the name. Data were analyzed using the MIXED and UNIVARIATE procedures in SAS version 9.4 (SAS Institute Inc., Cary, NC). Calves with birth weights ± 25% of the dairy's mean birth weight of 40.9 kg from 2018 to 2022 were excluded from the analysis. Five calves were not considered for analysis due to low birth BW of 25% below the average, and 1 calf was removed from postweaning BW and ADG due to its BW exceeding 25% of the average. Significance was declared at *P* < 0.05 and trends were discussed when 0.05 < *P* ≤ 0.10.

First-lactation performance can be influenced by early life vaccination status, feeding plans, and prevention of heat and cold stress (Mohd Nor et al., 2013; Chester-Jones et al., 2017). More emphasis should be placed on heat abatement and the microenvironment of the calf for them to reach their full potential as a member of the herd. According to Kohlman and Van Os (2020), the TNZ of a newborn calf is 10°C to 25°C, while a 30-d-old calf ranges from 0°C to 23°C. The mean average and maximum temperatures by season for July 2018 to February 2022 and TNZ are found in Table 1. Data from the National Weather Service's monthly averages for Urbana, Illinois, during the time of this analysis reported the mean average temperature for the summer as 23.5°C, 0.5°C above the TNZ for calves 30 d of age (National Oceanic and Atmospheric Administration, 2025). The maximum temperatures for those summers revealed a mean of 29.2°C, which is more than 4°C greater than the established TNZ for both calf age groups (National Oceanic and Atmospheric Administration, 2025). These temperature data suggest that calves raised during the summer months with limited heat mitigation experience some level of heat stress, with potentially diminishing performance.

**Table 1:** Mean average and maximum temperatures by season for July 2018 to February 2022

Season	Season^1^				Calf age	Thermal neutral zone^2^	
	Winter	Spring	Summer	Fall		Neonate	30 d
Mean average temperature, °C	−1.4	10.7	23.5	12.6	Thermal neutral zone, °C	10 to 25	0 to 23
Mean maximum temperature, °C	3.0	16.4	29.2	18.4			

1 Season: winter = December, January, February; spring = March, April, May; summer = June, July, August; fall = September, October, November.

2 Thermal neutral zone: ambient temperature range a calf can maintain stable body temperature without expending energy to heat up or cool down.

The average calf BW by season and contrasts of average BW are found in Table 2. Winter calves had greater birth (*P* = 0.002) and weaning (*P* < 0.001) BW when compared with summer calves. Birth BW of winter calves tended to be greater (*P* = 0.08) and weaning weights were greater (*P* < 0.001) when compared with all nonwinter calves. These observations agree with Hill et al., (2011) who observed improved ADG and feed efficiency for individually housed calves cooled with fans and shade compared with calves housed the same without fans. Similarly, it was reported that postnatal heat stress abatement moderates feed intake through weaning for calves cooled with fans and shade compared with those only provided shade (Dado-Senn et al., 2020). These studies observed greater intakes and ADG for cooled calves, suggesting similar results might be noted for calves during cooler seasons. Continuing the trend, summer calves had lower birth BW (*P* = 0.002) in contrast to all nonsummer calves, indicating potential for improved intake and ADG for calves during naturally cooler seasons. By weaning no difference was seen in the same contrast, further suggesting the impact of the warmest season may affect younger calves to a greater extent. Previous data reported that heat stress during the dry period of dairy cows decreased calf birth BW (Tao et al., 2012). Furthermore, Yaylak et al. (2015) observed that prenatal temperature-humidity index affected birth BW of calves, and increasing temperature-humidity index from 50 to 80 resulted in 3.8 kg decrease in birth BW, which could explain the differences in birth BW of the current analysis but is beyond the scope of this study. Inversely, postweaning BW were greater (*P* = 0.001) for summer calves in comparison to winter calves. Likewise, postweaning summer calves were observed to have greater BW (*P* = 0.04) in contrast to all nonsummer calves, whereas winter calves had lower BW (*P* = 0.003) when compared with all nonwinter calves. This agrees with Szabó et al. (2006) and their observations of summer calves having the greatest 205-d weights. Considering postweaning BW were measured when the calves were close to 6 mo of age, all summer-born calves would have been weighed in the winter, after experiencing the lower temperatures during cooler seasons. The excess energy used to maintain body temperature and possible reduction in intake to lower metabolic heat production could explain the differences in summer and winter BW.

**Table 2:** Average birth, weaning, and postweaning BW, and prewean, postwean, and pre- and postwean ADG for stages of weaning transition by birth seasons, and contrasts of interest focusing on winter and summer seasons

Item	Season^1^				SE	Contrast^2^ (*P*-value)		
	Winter	Spring	Summer	Fall		Winter vs. summer	Winter vs. nonwinter	Summer vs. nonsummer
BW^3^								
Birth, kg	40.8	41.4	38.4	39.4	0.14	0.002	0.08	0.002
Weaning, kg	90.6	79.9	83.3	84.0	0.35	<0.001	<0.001	0.26
Postweaning, kg	148.8	166.0	161.2	149.5	0.68	0.001	0.003	0.04
ADG^4^								
Prewean, kg/d	0.92	0.72	0.78	0.79	0.73	<0.001	<0.001	0.34
Postwean, kg/d	0.97	1.26	1.19	1.09	0.36	<0.001	<0.001	0.05
Pre- and postwean, kg/d	0.98	1.03	1.01	0.95	0.36	0.30	0.57	0.31

1 Season: winter = December, January, February; spring = March, April, May; summer = June, July, August; fall = September, October, November.

2 Contrasts: nonwinter = all months not included in winter; nonsummer = all months not included in summer.

3 BW at different stages: birth = d 0; weaning = d 56; postweaning = d 175.

4 AGD at different stages: prewean = 0 to 56 d; postwean = 57 to 175 d; pre- and postwean = 0 to 175 d.

Average daily gain of prewean, postwean, and the combination is in Table 2. Supporting the BW differences above, preweaning ADG of winter calves were greater than summer calves (*P* < 0.001) and all nonwinter calves (*P* < 0.001). This finding is in concurrence with previous research that stated dairy calves born in summer tended to have a lower ADG than those born in winter (Place et al., 1998). Further supporting the idea, other authors have stated that heat stress exerts a negative effect on the DMI, growth performance, health, and thermoregulatory responses of calves and heifers (Dado-Senn et al., 2020; Wang et al., 2020). Contrary to those data, the ADG of postweaning summer calves were greater when compared with winter calves (*P* < 0.001) and tended to be greater compared with all nonsummer calves (*P* = 0.05). Postweaning winter calves had a lower (*P* < 0.001) ADG in contrast to all nonwinter calves. This observation is thought to be possible due to the winter calves experiencing warmer temperatures from the summer season for the first time since the final weight was taken close to 6 mo of age. While winter-born calves often have greater DMI in the first months of life, once facing warmer weather they may decrease intake to lower metabolic heat production to help cool themselves. The mean average and maximum temperatures during the time of this analysis suggest potential for heat stress in the summer season compared with the TNZ (Table 1). This idea is supported by the observation by Hahn et al. (1990) that behavioral mechanisms such as reduction of feed intake help mature cattle cope with heat. Summer-born calves could hold back their full intake potential to regulate their body temperature, thus once cooler seasons approach, they have an easier time adjusting intake amounts, allowing for greater gains.

The average winter calf birth weights were 2.4 kg greater than summer-born calves, and summer calves were 2.1 kg less than nonsummer. That pattern continued through weaning, but reversed postweaning, where average BW for winter calves was 12.4 kg less than summer calves, and summer calves were 6.4 kg greater than nonsummer (Table 2). These contrasting trends resulted in combined ADG for the first 175 d of life that were not different among the 3 main contrasts (Table 2). Our findings coincide with remarks by Martin et al. (1962) that birth season effects were not a major source of variation, but extreme heat and cold can affect weight gain over short periods of time. They went on to add that the average conditions of all 4 seasons in their study were conducive to weight gain in young calves. With that, it is possible to consider the average environmental condition through the first 175 d to be an indicator of growth performance when feeding is not adjusted to meet the maintenance requirements of the calves.

Our results indicate that calf birth BW and growth performance are greater through weaning during colder seasons. Conversely, calves born during winter do not perform as well after weaning once the seasons get warmer. Summer calves were observed to have decreased birth BW and performance through weaning, but as the seasons became cooler, they excelled postweaning to 175 d of age. These data indicate a need for further research on the impacts of season on calf growth and efficiency, the effects of maternal heat stress during the dry period, and gestation length as possible lasting effects on future herd performance.
